# Dry pick-and-flip assembly of van der Waals heterostructures for microfocus angle-resolved photoemission spectroscopy

**DOI:** 10.1038/s41598-022-14845-z

**Published:** 2022-06-29

**Authors:** Satoru Masubuchi, Masato Sakano, Yuma Tanaka, Yusai Wakafuji, Takato Yamamoto, Shota Okazaki, Kenji Watanabe, Takashi Taniguchi, Jincai Li, Hirotaka Ejima, Takao Sasagawa, Kyoko Ishizaka, Tomoki Machida

**Affiliations:** 1 Institute of Industrial Science, The University of Tokyo, 4-6-1 Komaba, Meguro-ku, Tokyo, 153-8505 Japan; 2 Quantum-Phase Electronics Center and Department of Applied Physics, The University of Tokyo, Bunkyo-ku, Tokyo, 113-8656 Japan; 3 Materials and Structures Laboratory, Tokyo Institute of Technology, Yokohama, Kanagawa 226-8503 Japan; 4 Research Center for Functional Materials, National Institute for Materials Science, 1-1 Namiki, Tsukuba, 305-0044 Japan; 5 International Center for Materials Nanoarchitectonics, National Institute for Materials Science, 1-1 Namiki, Tsukuba, 305-0044 Japan; 6 Department of Materials Engineering, Graduate School of Engineering, The University of Tokyo, Bunkyo-ku, Tokyo, 113-8656 Japan; 7 RIKEN Center for Emergent Matter Science (CEMS), Wako, Saitama 351-0198 Japan

**Keywords:** Two-dimensional materials, Design, synthesis and processing

## Abstract

We present a dry pick-and-flip assembly technique for angle-resolved photoemission spectroscopy (ARPES) of van der Waals heterostructures. By combining Elvacite2552C acrylic resin and 1-ethyl-3-methylimidazolium ionic liquid, we prepared polymers with glass transition temperatures (*T*_g_) ranging from 37 to 100 ℃. The adhesion of the polymer to the 2D crystals was enhanced at ${T}_{\text{g}}$. By utilizing the difference in ${T}_{\text{g}}$, a 2D heterostructure can be transferred from a high-${T}_{\text{g}}$ polymer to a lower-${T}_{\text{g}}$ polymer, which enables flipping its surface upside down. This process is suitable for assembling heterostructures for ARPES, where the top capping layer should be monolayer graphene. The laser-based micro-focused ARPES measurements of 5-layer WTe_2_, 3-layer MoTe_2_, 2-layer WTe_2_/few-layer Cr_2_Ge_2_Te_6_, and twisted double bilayer WTe_2_ demonstrate that this process can be utilized as a versatile sample fabrication method for investigating the energy spectra of 2D heterostructures.

## Introduction

Two-dimensional van der Waals heterostructures provide unprecedented opportunities for exploring emergent correlated physics^1^. Angle-resolved photoemission spectroscopy (ARPES) is arguably the most direct tool for studying the electronic band structures of 2D van der Waals heterostructures^2^. However, studying such heterostructures by ARPES is challenging. To ensure the surface sensitivity requirement for ARPES, where most of the photoexcited electrons originate from the top few atomic layers^3^, a sample with an atomically flat and clean surface is required under ultrahigh vacuum conditions^2,4–10^. Covering a van der Waals heterostructure surface with monolayer graphene or hexagonal boron nitride (h-BN) helps meet this requirement^6^. The hydrophobic nature of graphene and h-BN surface allows the removal of the adsorbed contaminants by annealing under ultrahigh vacuum conditions. In addition, it allows the investigation of the energy spectra of heterostructures, where band structure hybridization with graphene and h-BN is absent. The photoexcited electrons can escape from the heterostructure without losing their momentum and energy.

An approach to fabricating van der Waals heterostructures for ARPES is the dry pick-up assembly^6,11–13^. Monolayer graphene is first picked up by a polymer (typically polycarbonate film on a polydimethylsiloxane block)^14^. Subsequently, the targeted 2D crystals are sequentially picked up by touching the monolayer graphene to the 2D crystals. Finally, the heterostructures were transferred onto a silicon substrate. Although these techniques allow the ARPES of WTe_2_^6^ and WSe_2_^11^, some challenges remain. This is because (i) the monolayer graphene on the polymer is torn after a few pick-up cycles, which prevents the assembly of heterostructures with multiple layers. (ii) The yield for picking up 2D flakes by monolayer graphene is lower than that in the case where thicker flakes are used. (iii) The process is incompatible with the tear-and-stack method for fabricating twisted heterostructures with precise rotational angle control, as it requires a thick 2D flake to be placed on the pick-up polymer^15^. Therefore, there is demand for developing a method to assemble heterostructures in reverse order, that is, from thick flakes to thin flakes, and flip their surface upside down and drop it onto a designated substrate, which we call a pick-and-flip assembly technique. So far, several pick-and-flip assembly techniques have been developed to fabricate van der Waals heterostructures for STM measreuments, however these techniques are incompatibie with the convenrional glovebox enclosure because they use water^16,17^ or orgnic solvents^18^ to transfer the heteroctructures from one polymer stamp to the other second stamp.

**Figure 1:**
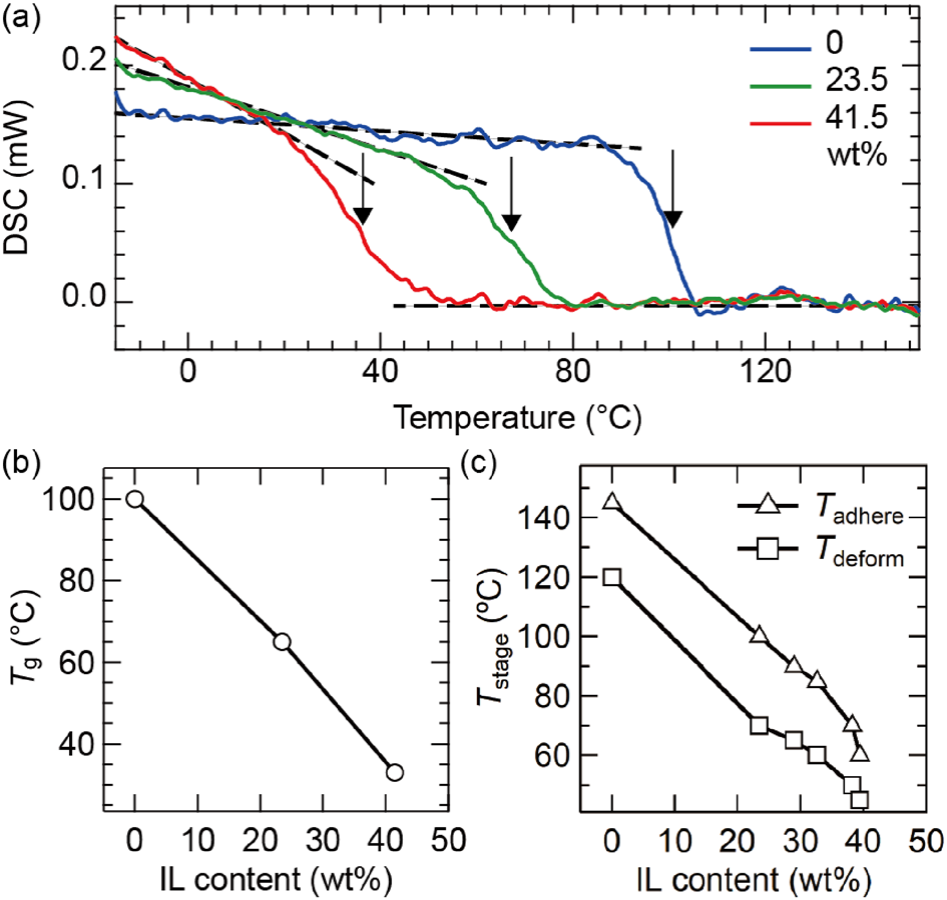
(**a**) Differential scanning calorimetry curves of Elvacite2552C-IL compounds for varying IL contents (blue) 0, (green) 23.5, and (red) 41.5 wt%. (**b**) Glass transition temperature $\left({T}_{\text{g}}\right)$ of Elvacite2552C-IL compounds as a function of the IL content. (**c**) Stage temperature $\left({T}_{\text{stage}}\right)$ at which the Elvacite2552C-IL compounds deform (${T}_{\text{deform}}$) while contacting with the silicon substrate and adhere $({T}_{\text{adhere}})$ to the silicon substrate for varying IL contents.

In this letter, we present a dry pick-and-flip assembly technique for the ARPES of van der Waals heterostructures by employing the differences in the glass transition temperatures of a polymer composed of Elvacite2552C acrylic resin and 1-ethyl-3-methylimidazolium ionic liquid (IL). First, we characterized the thermal properties of Elvacite2552C–IL compounds. Figure 1a shows their differential scanning calorimetry (DSC) for different IL contents (red) 0, (blue) 23.5, and (green) 41.5 wt%. For all the IL contents, the DSC curves exhibit step-like transitions, indicating an increase in the heat capacities [black dashed lines]. This indicates that Elvacite2552C-IL exhibits a transition from a brittle state to a plastic state, that is, glass transition. The temperature at the middle of the sloped region is taken as the ${T}_{\text{g}}$ [black arrows in Fig. 1a]. ${T}_{\text{g}}$ decreases to 100, 70, and 37 ℃ when increasing the IL content to 0, 23.5, and 41.5 wt% (Fig. 1b). Next, we evaluated the mechanical properties of the Elvacite2552C-IL compounds. Here, we investigated the changes in the shape of the Elvacite2552C-IL compounds on a glass slide after touching and detaching them onto the silicon substrate by varying the sample stage temperature ${T}_{\text{stage}}$. When ${T}_{\text{stage}}$ is low, the shape of the Elvacite2552C-IL compounds is unaffected (Supplementary Fig. S1b-(i)). When ${T}_{\text{stage}}$ is increased, they deform after detaching from the silicon substrate (Supplementary Fig. S1b-(ii)). We define ${T}_{\text{stage}}$ at which Elvacite2552C-IL compounds undergo deformation as ${T}_{\text{deform}}$. With further increase in ${T}_{\text{stage}}$, the Elvacite2552C-IL compounds strongly adhere to the silicon substrates. The polymer residues remained on the silicon substrate (Supplementary Fig. S1b-(iii)). We define ${T}_{\text{stage}}$ at which Elvacite2552C-IL compounds start adhesion as ${T}_{\text{adhere}}$. As shown in Fig. 1c, ${T}_{\text{deform}}$ and ${T}_{\text{adhere}}$ systematically decrease from ${T}_{\text{deform}}=120 ^\circ \text{C}$ to $45^\circ \text{C}$ (squares in Fig. 1c) and ${T}_{\text{adhere}}=145$–$60^\circ \text{C}$ (triangles in Fig. 1c) when increasing the IL content from 0 to 39.4 wt%. These results indicate that the IL functions as a volatile plasticizer for Elvacite2552C, and ${T}_{\text{g}}$ of Elvacite2552C can be systematically controlled using the IL content.

**Figure 2:**
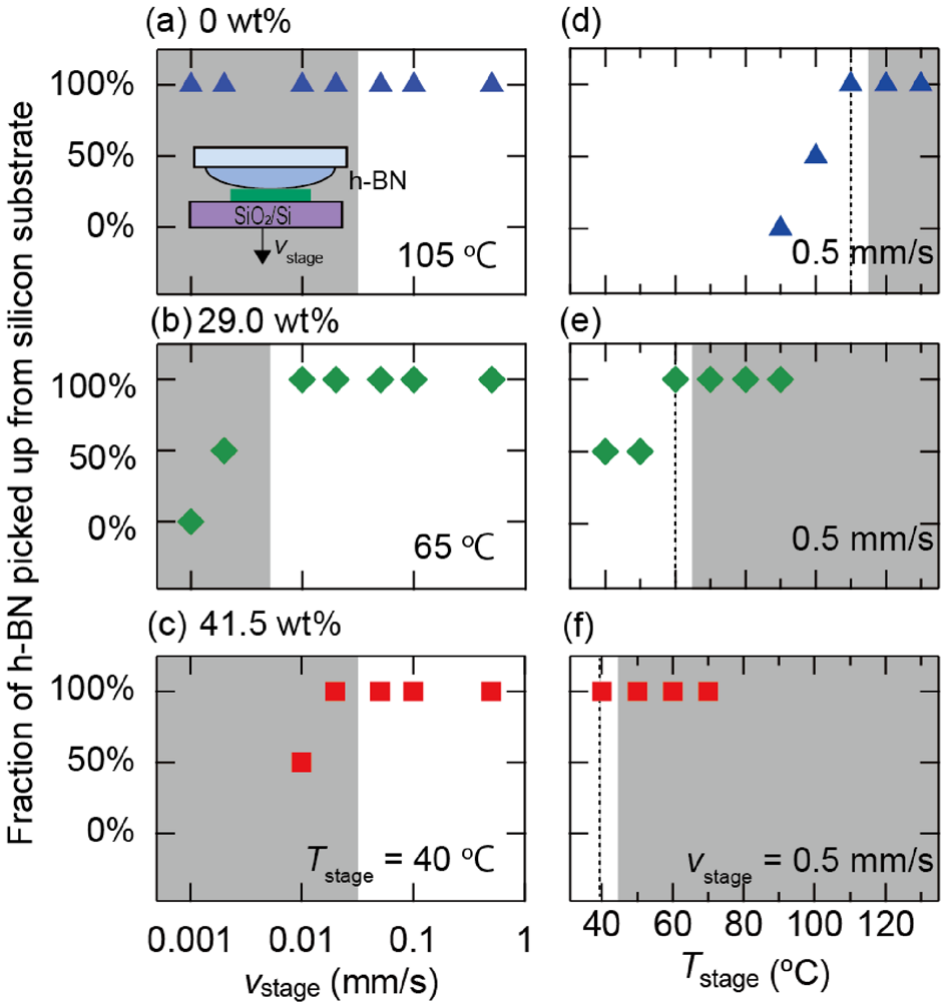
(**a**–**c**) Fraction of h-BN flakes picked up by Elvacite2552C-IL compounds from a silicon substrate with different speeds $\left({v}_{\text{stage}}\right)$ to detach the silicon substrate from the compounds for IL contents (a) $0$, (b) $29.0$, and (**c**) $41.5$ wt% at stage temperatures (**a**) ${T}_{\text{stage}}=105$, (**b**) $65$, and (**c**) $40^\circ{\rm C}$. (**d**–**f**) Fraction of h-BN flakes transferred from the silicon substrate to Elvacite2552C-IL compounds for varying stage temperatures $\left({T}_{\text{stage}}\right)$ at ${v}_{\text{stage}}=0.5 \text{mm}/\text{s}$. The gray shaded regions indicate the ranges of ${{v}_{\text{stage}} \text{and }T}_{\text{stage}}$, where the Elvacite2552C-IL compounds are substantially deformed while picking up the h-BN flakes and do not retain their original dome shape [“Supplementary Information”].

Next, we investigated the adhesion of the Elvacite2552C-IL compounds to 2D flakes. Here, we selected h-BN flakes on a SiO_2_/Si substrate to represent 2D flakes and tested whether the Elvacite2552C-IL compound can pick up the h-BN flakes (inset in Fig. 2a). Figure 2a–c show the fraction of h-BN flakes picked up by the Elvacite2552C-IL compounds when varying the stage speed $\left({v}_{\text{stage}}\right)$ to detach the silicon substrate from the compounds. Here, ${v}_{\text{stage}}$ was precisely controlled using an actuator with stepper motors. The IL contents were (a) 0, (b) 29.0, and (c) 41.5 wt%. The stage temperatures were close to ${T}_{\text{g}}$ of each Elvacite-IL compound: (a) ${T}_{\text{stage}}=105, \left(\text{b}\right) 65,$ and (c) 40 °C. For IL content of 0 wt% and ${v}_{\text{stage}}=0.001$–0.02 mm/s, the polymer strongly adhered to the silicon substrate. The polymer shape was substantially deformed [representative optical microscope images and schematics are shown in Supplementary Fig. S2]. Substantial deformation prevents the polymer from further picking up the 2D crystals. Therefore, these conditions are unsuitable for assembling van der Waals heterostructures [indicated by the dark shaded region in Fig. 2a]. When the stage speed is increased in the range of 0.05–0.5 mm/s, the extent of deformation reduced, and h-BN was successfully transferred onto the polymer (Fig. 2a). This indicates an increase in the elastic modulus of the polymer for higher ${v}_{\text{stage}}$ i.e., the stretching speeds applied to the polymer. The same trends can be observed for IL contents of 29.0 wt% (Fig. 2b) and 41.5 wt% (Fig. 2c). These results indicate that a higher ${v}_{\text{stage}}$ is suitable for utilizing the Elvacite2552C-IL compound to pick up the 2D flakes.

Based on the observations described above, we fixed the stage speeds to the highest value achievable with our stepping motor stage ${v}_{\text{stage}}=0.5 \text{ mm}/\text{s}$ and investigated the influence of varying ${T}_{\text{stage}}$. Figure 2d–f show the fraction of h-BN flakes picked up by the Elvacite2552C-IL compound as a function of ${T}_{\text{stage}}$ for varying IL contents. For an IL content of 0 wt%, the h-BN flakes were not transferred to the Elvacite2552C-IL compound at $T{}_{\text{stage}}<100^\circ{\rm C}$. At ${T}_{\text{stage}}=100^\circ{\rm C}$, approximately half of the h-BN flakes were transferred to the polymer. At $T{}_{\text{stage}}=110^\circ{\rm C}$, the h-BN flakes were fully transferred to the polymer. With further increase in ${T}_{\text{stage}}$, the polymer exhibited substantial deformation, similar to that observed in low ${v}_{\text{stage}}$ cases. Therefore, ${T}_{\text{stage}}=100^\circ{\rm C}$ is suitable when using Elvacite2552C-IL compound with an IL content of 0 wt% for picking up h-BN flakes. When the IL content was increased to 29.0 wt% and 41.5 wt%, ${T}_{\text{g}}$ of the Elvacite2552C-IL compound decreased. The optimum ${T}_{\text{stage}}$, where the h-BN flakes were transferred to the polymer, shifted to lower temperatures ${T}_{\text{stage}}=60^\circ{\rm C}$ and $40^\circ{\rm C}$ (Fig. 2e,f). These results indicate that a ${T}_{\text{stage}}$ value close to ${T}_{\text{g}}$ is suitable for picking up h-BN flakes from silicon substrates.

These results show that ${T}_{\text{stage}}$ required to pick-up h-BN flakes can be continuously controlled between 110 and 40 ℃ by utilizing the Elvacite2552C-IL compounds. So far, various polymers have been utilized for picking up 2D flakes from silicon substrates^19^, including polypropylene carbonate $\left({T}_{\text{g}}\sim 40^\circ \text{C}\right)$^20^, Elvacite2552C $\left({T}_{\text{g}}\sim 100^\circ \text{C}\right)$^21^, and polycarbonate $\left({T}_{\text{g}}\sim 140^\circ \text{C}\right)$^14^. In contrast, ${T}_{\text{g}}$ of the Elvacite2552C-IL compound can be controlled between 40 and 100 °C, providing a wider range of substrate temperatures to assemble van der Waals heterostructures. Previously, the glass transition temperatures of poly(methyl methacrylate)^22,23^ and poly (vinyl chloride)^24^ could be controlled by adding ILs. Therefore, ILs would be utilized for tuning the ${T}_{\text{stage}}$ required to pick-up 2D flakes in much wider range, by combining with the other polymers such as polypropylene carbonate and polycarbonate.

**Figure 3:**
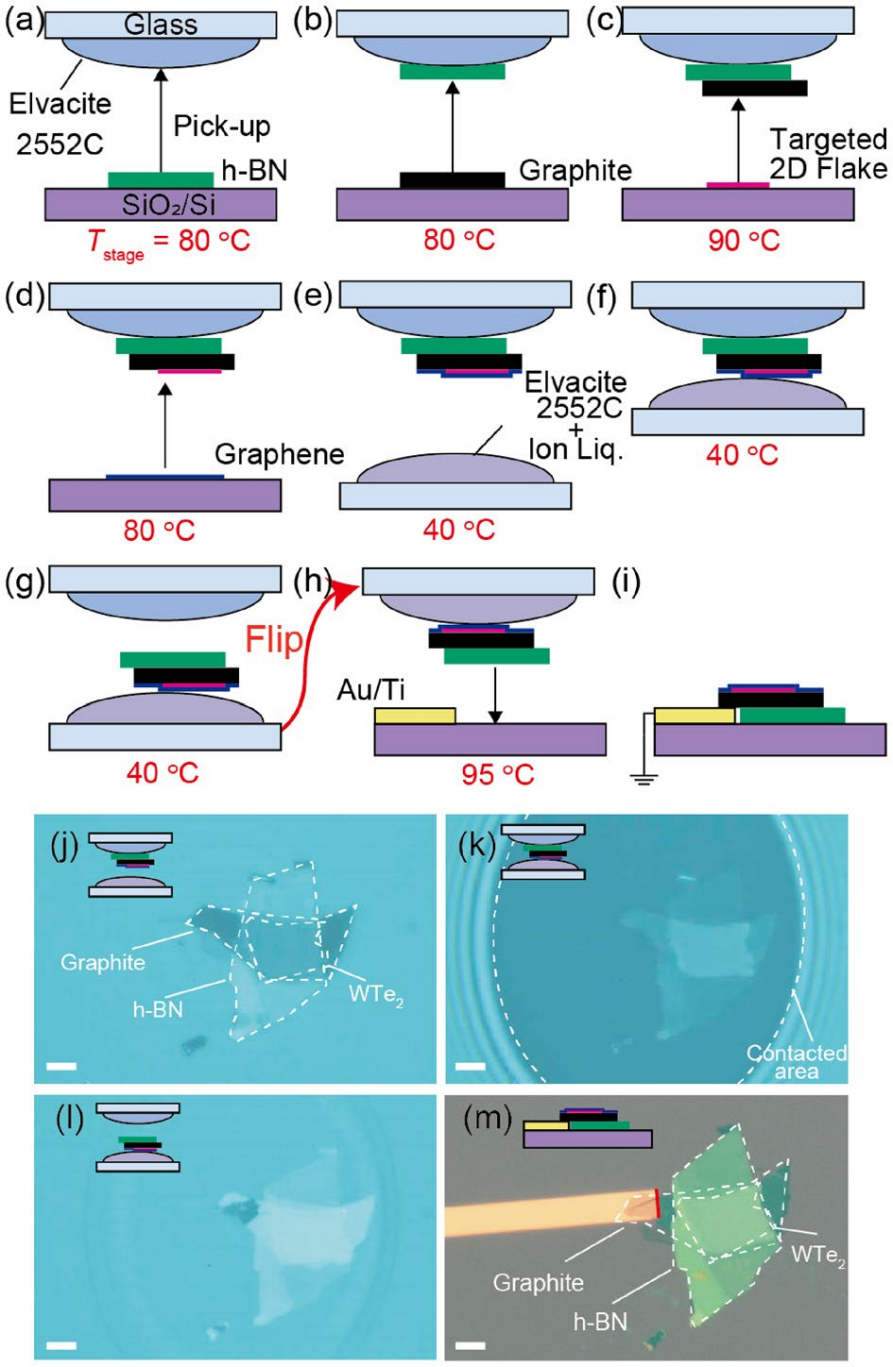
Schematics of dry pick-and-flip assembly process of 2D crystals for ARPES. First, (**a**) thick h-BN, (**b**) thick graphite, (**c**) targeted 2D crystals, and (**d**) graphene are picked-up by Elvacite2552C at ${T}_{\text{stage}}$ of (**a**) $80$, (**b**) $80$, (**c**) $90$, and (**d**) $80^\circ \text{C}$ inside the glovebox. (**e**–**g**) Heterostructure on Elvacite2552C is released onto a second Elvacite2552C-IL compound with an IL content of 39.4 wt% at ${T}_{\text{stage}}=40^\circ \text{C}$. The adhesive force of Elvacite2552C to the heterostructure is weaker than to that of Elvacite2552C-IL compound; the heterostructure transfers to the second stamp. (**g**) and (**h**) Heterostructure is flipped and released onto a SiO_2_/Si substrate with prepatterned metal electrode in the ambient condition at $T=95^\circ \text{C}$. (**j**–**m**) Optical microscopic images of the heterostructure corresponding to (**e**–**i**). The scale bars correspond to 10 ${\mu}{\text{m}}$.

By utilizing the difference in ${T}_{\text{g}}$ of Elvacite2552C-IL compounds with varying IL contents, we can develop a process to assemble van der Waals heterostructures for ARPES, as schematically shown in Fig. 3a-i [Optical microscopic images of the heterostructure corresponding to (e–i) are shown in Fig. 3j–m, respectively]. The assembly was conducted in a glovebox enclosure^21^. First, a thick h-BN flake was picked up by Elvacite2552C without IL on a glass slide at ${T}_{\text{stage}} = 80^\circ \text{C}$ (Fig. 3a). The thick graphite was picked up by h-BN (Fig. 3b). The targeted 2D crystal for the ARPES study was picked up by graphite at ${T}_{\text{stage}} = 90^\circ \text{C}$ (Fig. 3c). Finally, the targeted 2D crystal was covered by picking up monolayer graphene at $T = 80^\circ \text{C}$(Fig. 3d). The temperature was lowered to ${T}_{\text{stage}} = 40^\circ \text{C}$, and the heterostructure touched Elvacite 2552C-IL compound with an IL content of 39.4 wt% (Fig. 3f). Because the adhesion of the heterostructure to Elvacite2552C-IL compound is stronger than that to Elvacite2552C at ${T}_{\text{stage}} = 40^\circ \text{C}$, the heterostructure is transferred to the Elvacite2552C + IL compound surface (Fig. 3g). The heterostructure was released onto the SiO_2_/Si substrate with a metal electrode (Au/Ti) at $T = 95^\circ \text{C}$ in the ambient condition. Finally, the Elvacite 2552C-IL  compound was removed by immersing the substrate in chloroform for 1 min. Through this process, the heterostructure surface was flipped upside down.

To confirm that the van der Waals heterostructures fabricated using the presented pick-and-flip assembly method have sufficiently clean surfaces for ARPES measurements, we fabricated various 2D heterostructures and conducted laser-based micro-focus ARPES (µ-ARPES) measurements^25^. The samples were mounted onto the ARPES stage with silver paste to form an electrical connection to the metal electrode. The samples were transferred to the ultrahigh vacuum chamber of the ARPES system using the ICF-70 nipple chamber with a gate valve, which was utilized as a simple vacuum suitcase pumped to ${10}^{-3}$ Pa. Before the measurement, the samples were annealed at approximately 200 °C for ~ 10 h under ultrahigh vacuum.

**Figure 4:**
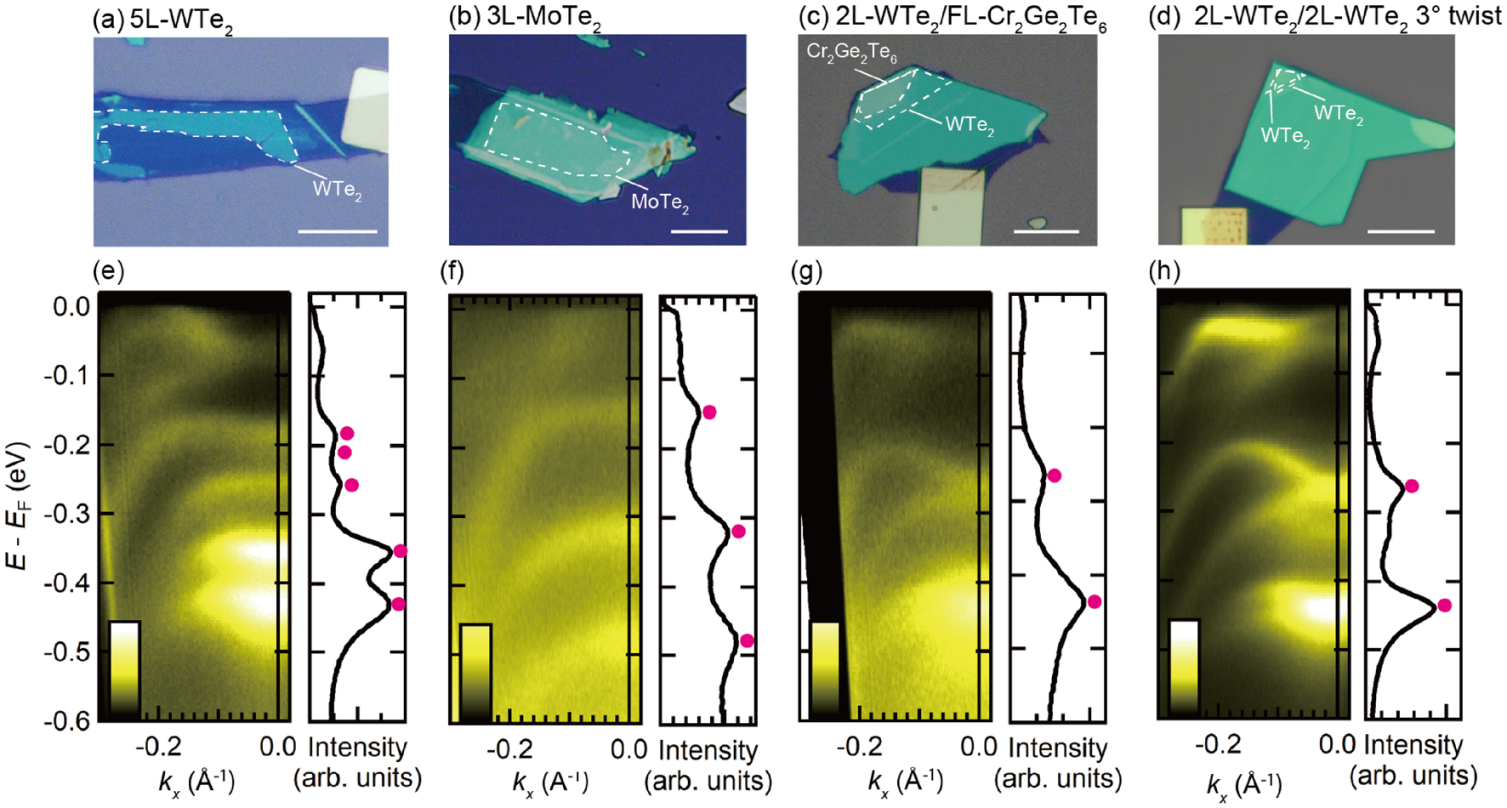
(Upper panels) Optical microscopic images of (**a**) 5-layer WTe_2_, (**b**) 3-layer MoTe_2_, (**c**) 2-layer WTe_2_/few-layer Cr_2_Ge_2_Te_6_, and (**d**) twisted double 2-layer WTe_2_ with a rotation angle of $3^\circ$. These flakes are encapsulated between monolayer graphene and graphite. The white scale bar corresponds to 10 ${\mu}{\text{m}}.$ (Lower panels) The ARPES images of (**e**) 5-layer WTe_2_, (**f**) 3-layer MoTe_2_, (**g**) 2-layer WTe_2_/few-layer Cr_2_Ge_2_Te_6_, and (**h**) twisted double 2-layer WTe_2_ recorded along the ${k}_{x}$ direction. For the twisted double 2-layer WTe_2_, the *k*_*x*_ axis is set to the direction tilted ± 1.5 degrees from the *k*_*x*_ axes of the top and bottom 2-layer WTe_2_. Energy distribution curves at ${k}_{x}=0$ obtained with an integral width of 0.04/Å$.$ The red markers indicate the positions of the intensity peaks derived from the topmost WTe_2_ or MoTe_2_ flakes.

Figure 4 shows the optical microscope images of (a) 5-layer WTe_2_, (b) 3-layer MoTe_2_, (c) 2-layer WTe_2_/Cr_2_Ge_2_Te_6_, and (d) twisted double bilayer WTe_2_, which are representatives of the heterostructures fabricated using the assembly method. Figure 4e–h show the ARPES images taken of each sample. In Fig. 4e, we can observe the well-resolved energy band structure of 5-layer WTe_2_, comprising five sets of distinct energy spectra. It well demonstrates that the discrete band dispersions obtained by the confinement effect due to the finite number of stacking layers are clearly observed. The layer-number dependence of the energy spectrum of exfoliated WTe_2_ flakes can be investigated using this method^25^. These observations indicate that even easily oxidizable materials can be assembled using this method, providing a sufficiently clean interface between graphene and WTe_2_ and the formation of a monolayer graphene surface, enabling the direct observation of the band structures of exfoliated transition metal dichalcogenides. Figure 4f shows the ARPES data from 3-layer MoTe_2_, which resolved the semimetallic energy spectrum of MoTe_2_, indicating the applicability of the fabrication technique to different materials. The band structure of 2-layer WTe_2_ on a magnetic thin flake Cr_2_Ge_2_Te_6_ could be measured by µ-ARPES (Fig. 4g), indicating the applicability of the fabrication technique to study composite van der Waals heterostructures. Finally, the ARPES data from the twisted double bilayer WTe_2_ could be used to resolve a well-separated energy spectrum (Fig. 4h); the observed spectrum is distinct from those of the 2-layer WTe_2_ and 4-layer WTe_2_, indicating the signature of band structure hybridization in the twisted WTe_2_ heterostructure. This result demonstrates that the proposed method is compatible with the tear-and-stack technique to assemble twisted van der Waals heterostructures.

We described a dry pick-and-flip assembly technique for the ARPES of van der Waals heterostructures by employing the differences in ${T}_{\text{g}}$ of a polymer composed of Elvacite2552C and an IL with differing IL contents. The observations of the ARPES spectrum of various heterostructures indicate that the method can be utilized to obtain van der Waals heterostructures with a sufficiently clean surface under ultrahigh vacuum conditions. The fabrication technique allows us to assemble van der Waals heterostructures from thick flakes to thin flakes and flip their surface upside down and drop it onto a designated substrate. The presented technique can be utilized as a versatile sample fabrication method to investigate the energy spectrum of various van der Waals heterostructures.

## Methods

### Preparation of Elvacite2552C-IL compounds

A thermoplastic methacrylate copolymer (Elvacite2552C, Lucite International) powder and an ionic liquid (1-ethyl-3-methylimidazolium bis (trifluoromethylsulfonyl)imide, Iolitec GmbH), were mixed in a screw-tube bottle. Anisole was added to the mixture at a volume ratio of 1:1. The mixture was left in the cabinet for five days to dissolve Elvacite2552C-IL mixture to anisole. A droplet of Elvacite2552C, IL, and anisole solution was formed on the glass slide using a needle. The glass slide was baked at $180^\circ{\rm C}$ in a vacuum oven for > 12 h or placed on a hotplate at $180^\circ{\rm C}$ inside a glovebox for > 8 h to evaporate the anisole solvent.

### DSC measurements

DSC measurements were performed using a Shimadzu DSC-60 Plus. The Elvacite2552C-IL-Aisole solution was cast onto a DSC pan and baked in a vacuum oven at $180^\circ{\rm C}$ for 15 h to evaporate the anisole solvent. Elvacite2552C-IL compounds weighing approximately 5 mg were packed into an aluminum crimp pan. The DSC curves were measured at a temperature ramp speed of $10^\circ{\rm C} /\text{min}$.

### Laser-based micro-focused angle-resolved photoemission spectroscopy (μ-ARPES)

The laser-based µ-ARPES measurement was performed by using a combination of the hemispherical analyzer (DA30, Scienta Omicron Inc.) and the fourth-harmonic generation with photon energy $h\nu =6.42 \text{ eV}$ of Ti:sapphire laser radiation (Verdi V-18 and MIRA-HP, Coherent Inc.) obtained by the frequency convertor (HarmoniXX, APE Inc.). The laser incident light was focused by the optical lens system^26^ (NTT Advanced Technology corporation) equipped outside with the ultra-high vacuum chamber of the ARPES. The spot size is estimated to be approximately 20 μm. The total energy resolution was set to 3 meV. During measurement, a sample manipulator temperature was kept below 20 K. All ARPES images in this article represent the sums of the ARPES intensities taken with the *s*- and *p*-polarized light.

## Supplementary Information

Supplementary Information.

## Data Availability

The data that support the findings of this study are available from the corresponding author upon reasonable request.
