# Supplementary material for: Dry pick-and-flip assembly of van der Waals heterostructures for microfocus angle-resolved photoemission spectroscopy
Source: Sci Rep. 2022 Jun 29;12:10936. doi: 10.1038/s41598-022-14845-z (PMC9243021; doi:10.1038/s41598-022-14845-z)
Supplement: Supplementary file 1 — Supplementary Information. [file 41598_2022_14845_MOESM1_ESM.pdf]

# (Supplementary material)

# Dry pick-and-flip assembly of van der Waals heterostructures for microfocus angle-resolved photoemission spectroscopy


Satoru Masubuchi[1,†,*], Masato Sakano[2,†], Yuma Tanaka[2,†], Yusai Wakafuji[1], Takato Yamamoto[2], Shota Okazaki[3], Kenji Watanabe[4], Takashi Taniguchi[1,5], Jincai Li[6], Hirotaka Ejima[6], Takao Sasagawa[3], Kyoko Ishizaka[2,7], and Tomoki Machida[1,*]

[1] *Institute of Industrial Science, University of Tokyo, 4-6-1 Komaba, Meguro-ku, Tokyo 153-8505, Japan*

[2] *Quantum-Phase Electronics Center and Department of Applied Physics, The University of Tokyo, Bunkyo-ku, Tokyo 113-8656, Japan*

[3] *Materials and Structures Laboratory, Tokyo Institute of Technology, Yokohama, Kanagawa 226-8503, Japan*

[4] *Research Center for Functional Materials, National Institute for Materials Science, 1-1 Namiki, Tsukuba 305-0044, Japan*

[5] *International Center for Materials Nanoarchitectonics, National Institute for Materials Science, 1-1 Namiki, Tsukuba 305-0044, Japan*

[6] *Department of Materials Engineering, Graduate School of Engineering, The University of Tokyo, Bunkyo-ku, Tokyo 113-8656, Japan*

[7] *RIKEN Center for Emergent Matter Science (CEMS), Wako, Saitama, 351-0198, Japan*

[†] *These authors contributed equally to this work.*

*Correspondence: msatoru@iis.u-tokyo.ac.jp, tmachida@iis.u-tokyo.ac.jp

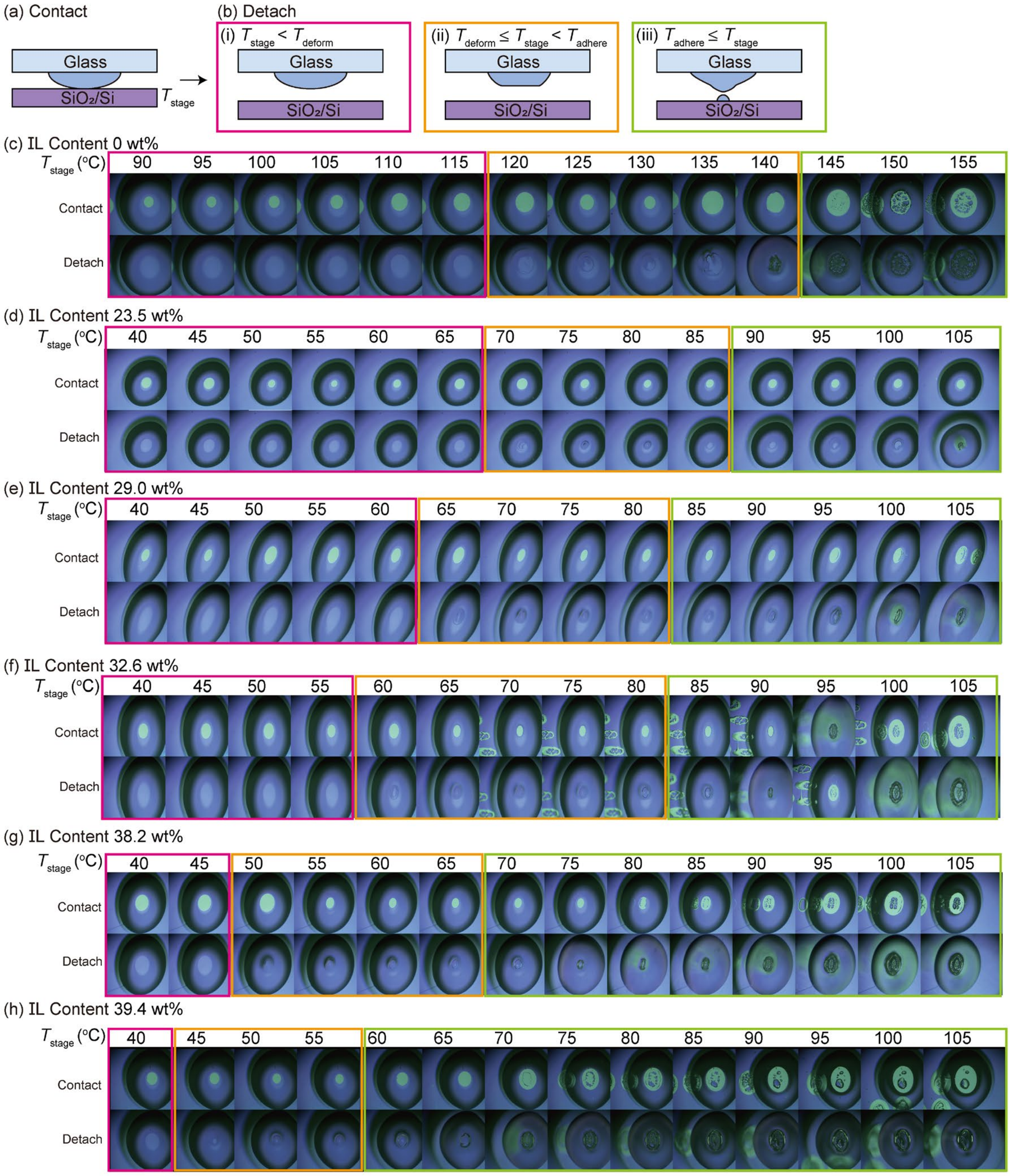


Supplementary Figure 1. Measurement of deformation ($T_{\mathrm{deform}}$) and adhesion temperature ($T_{\mathrm{adhere}}$) of Elvacite2552C-IL compounds. (a) Schematics of Elvacite2552C-IL compound contacting with the silicon substrate. (b) Schematics of Elvacite 2552C-IL compound after detaching from the silicon substrate. (i) Elvacite2552-IL compound is not deformed for $T_{\mathrm{stage}} < T_{\mathrm{deform}}$. (ii) Elvacite2552C-IL compound is deformed for $T_{\mathrm{deform}} \leq T_{\mathrm{stage}} < T_{\mathrm{adhere}}$. (iii) Elvacite2552C-IL compound is adhered to the silicon substrate i.e., a portion of the

Elvacite2552C-IL compound remains on the surface of the silicon substrate at $T_{\text{adhere}} \leq T_{\text{stage}}$. (c)-(h) Optical microscope images of Elvacite2552C-IL compounds while contacting with the silicon substrate (upper rows) and after detaching from the silicon substrate (bottom rows). The ionic liquid contents are varied as (c) 0, (d) 23.5, (e) 29.0, (f) 32.6, (g) 38.2, and (h) 39.4 wt%. The magenta, orange, and green rectangles indicate the ranges of $T_{\text{stage}}$ corresponding to the schematics shown in (b)-(i), (ii), and (iii), respectively.

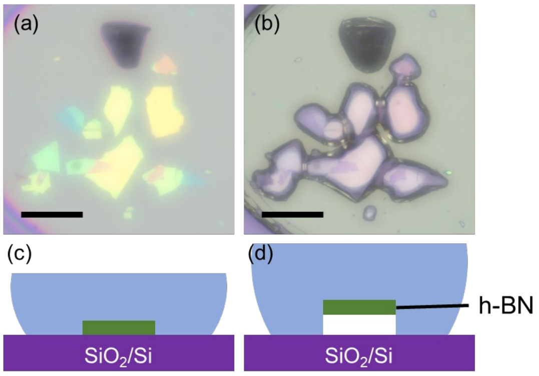


Supplementary Figures 2. (a) and (b) Representative optical microscope images of Elvacite2552C-IL compound, where it is substantially deformed while detaching from the silicon substrate. (a) During contact with the silicon substrate and (b) after detachment from the silicon substrate. (c) and (d) schematics of the polymer/h-BN/silicon substrate corresponding to the cases shown in (a) and (b), respectively. The polymer is Elvacite2552C without an ionic liquid. The temperature stage is $T_{\mathrm{stage}} = 110\ ^\circ\mathrm{C}$. The speed to detach the silicon substrate from Elvacite2552C is $v_{\mathrm{stage}} = 0.01\ \mathrm{mm/s}$.

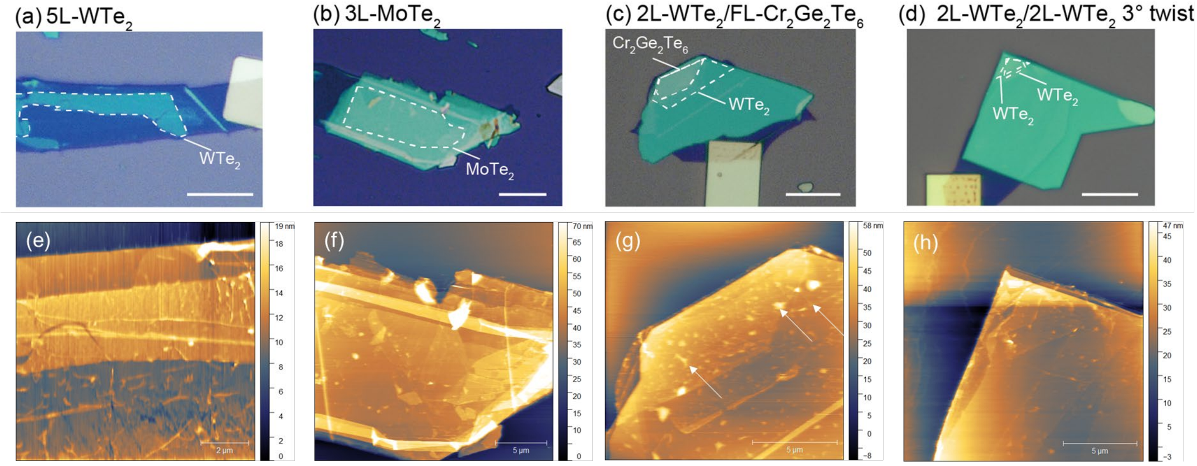


Supplementary Figure 3. (a)-(d) optical microscopic images of (a) 5-layer $WTe_2$, (b) 3-layer $MoTe_2$, (c) 2-layer $WTe_2$/few-layer $Cr_2Ge_2Te_6$, and (d) twisted double 2-layer $WTe_2$ with a rotation angle of 3°, presented in Fig. 4 of main paper. The white scale bars correspond to 10 μm. (e)-(h) Atomic force microscope images of (e) 5-layer $WTe_2$, (f) 3-layer $MoTe_2$, (g) 2-layer $WTe_2$/few-layer $Cr_2Ge_2Te_6$, and (h) twisted double 2-layer $WTe_2$.

In all topography images, we find some bubbles/wrinkles in the capping graphene layer [as indicated by white arrows in (e)]. However, the encapsulated TMD flakes keep their original shapes as it was on the silicon substrates. No noticeable cracks formed during the assembly process. We also observe some surface residues in (a), the actual surface cleanness in the ARPES measurement chamber is much better than those indicated in the AFM images because the sample was annealed at 200 ℃ for 10 hours before conducting ARPES measurements, and we conducted the AFM measurements in the ambient condition.
